# Antiviral activity of *Bifidobacterium adolescentis *SPM1005-A on human papillomavirus type 16

**DOI:** 10.1186/1741-7015-10-72

**Published:** 2012-07-12

**Authors:** Min-Kyeong Cha, Do-Kyung Lee, Hyang-Mi An, Si-Won Lee, Seon-Hee Shin, Jeong-Hyun Kwon, Kyung-Jae Kim, Nam-Joo Ha

**Affiliations:** 1 College of Pharmacy, Sahmyook University, Seoul, Republic of Korea; 2 Department of Pediatrics, College of Medicine, Hallym University, Chuncheon, Republic of Korea; 3 Department of Sport Medicine, Jung Won University, Chungbuk, Republic of Korea

**Keywords:** antiviral activity, *Bifidobacterium adolescentis *SPM1005-A, human papillomavirus (HPV) type 16, quantitative real-time PCR (qRT-PCR)

## Abstract

**Background:**

Probiotic lactic acid bacteria (LAB) support a functional and balanced immune system, and contribute to immune modulatory effects in combatting microbial pathogens, including viruses. Most cervical cancers are associated with anogenital region infection with high-risk (HR) human papillomavirus (HPV). In this study, we analyzed the antiviral activity of *Bifidobacterium adolescentis *SPM1005-A in the SiHa cervical cancer cell line expressing HPV type 16.

**Methods:**

We assessed the cellular toxicity of *B. adolescentis *SPM1005-A in SiHa cells by the Trypan blue dye exclusion assay. Cells (3.6 × 10^5^) in culture plates with or without *B. adolescentis *SPM1005-A in the same type of medium, were incubated with HPV type 16 at a concentration of 5.1 × 10^7 ^cfu/ml. For antiviral analysis, we performed quantitative real-time PCR (qRT-PCR) for E6 and E7 oncogene expressions and observed protein levels by immunoblotting.

**Results:**

The qRT-PCR results showed that E6 and E7 mRNA levels decreased simultaneously. Western blot analysis revealed that the E6 protein expression slightly decreased after 24 and 48 h, but the level of E7 protein expression appear unaffected compared with that in the control. Decreased HPV16 E6 and E7 mRNA transcript and protein levels were not associated with cell morphology or significant cytotoxic effects.

**Conclusions:**

This study showed that *B. adolescentis *SPM1005-A had antiviral activity through suppression E6 and E7 oncogene expression. The results suggest that *B. adolescentis *SPM1005-A could be potential applications of HPV-associated cervical cancer prevention.

## Background

Cervical cancer is the second most common malignant disease of the female reproductive organs, with an incidence per year of almost half a million and a mortality rate of approximately 25% [1]. Most cervical cancers are associated with the anogenital region or mucosa cell infection with human papillomavirus (HPV) [2]. Of the more than 200 different HPV types identified, 30 HPV types infect the anogenital skin and oral mucosa and can be further classified as low risk (LR) or high risk (HR) based on the clinical prognosis of their associated lesions [3]. Approximately 99.7% of cervical cancers contain viral DNA of HR types, with type 16 being the most prevalent, followed by types 18, 31, 33 and 45 [4]. The malignant phenotype of HR types depends on the expression of two viral genes E6 and E7, which bind to p53 and retinoblastoma protein (pRb) and neutralize their function, respectively [5]. The most important function of E6 protein is binding of the tumor suppressor p53, which leads to it degradation through an ubiquitin proteolytic pathway. Degradation of p53 bypasses the normal growth arrest signals at the G1/S and G2/M checkpoints and is the major cause of chromosomal instability, with mutational consequences for HPV-positive cells [6]. The E7 protein interacts with pRb and releases transcription factor E2F, which induces expression of genes involved in cellular differentiation and proliferation [7,8]. Therefore, the studies for inhibitors of the oncogenic proteins E6 and E7 of HPV type 16 are constantly in progress.

Lactic acid bacteria (LAB) are widely used and generally recognized as safe organisms for animal and human applications. They produce antimicrobial substances such as organic acids, hydrogen peroxide, diacetyl and bacteriocins, which have beneficial effects on the host organisms [9]. Probiotic LAB support functional and balanced immune systems and contribute to immune modulatory effects in combatting microbial pathogens, including viruses [10]. Several studies have reported that LAB such as *Lactobacilli *boost the antiviral effect against human rotaviruses that cause diarrhea, human immunodeficiency virus type 1 and influenza virus [11-13].

Among commensal bacteria, *Bifidobacteria *is one of the most numerous probiotics in the mammalian gut that belong to LAB [14]. Xiao *et al. *have reported cholesterol reduction by a supplement containing *Bifidobacterium longum*, and Le Leu *et al. *reported the potential of *Bifidobacterium animalis *subspecies *Lactis *to prevent colorectal cancer. Also, antitumor activity has been studied in peptidoglycans isolated from a *Bifidobacterium infantis *strain [15-17]. Despite the various literatures indicating a protective effect of *Bifidobacteria *in epidemiological studies, the antiviral effects have not yet been studied in detail. We therefore assessed the antiviral activity of *B. adolescentis *SPM1005-A on E6 and E7 mRNA transcript and protein levels in the SiHa cervical cancer cell line expressing HPV type 16 *in vitro*.

## Methods

### Preparation of *B. adolescentis *SPM1005-A

For the isolation of *Bifidobacteria*, fecal samples were collected from healthy Koreans (aged 20 to 30 years old) by BD BBLanaerobic sample collection and transport system (Becton Dickinson and Co, USA) to maintain anaerobic conditions. Fecal samples were serially diluted tenfold from 10^-1 ^to 10^-8^, and 100 μl were spread into selective blood liver agar (Nissui Pharm, Japan) containing 5% sheep blood. After 48 h of incubation in anaerobic conditions (90% N_2_, 5% H_2_, 5% CO_2_) (Bactron Anaerobic Chamber, USA) at 37°C, brown or reddish-brown colonies 2 mm to 3 mm in diameter were selected for further identification [18]. A fructose-6-phosphate phosphoketolase (F6PPK) test was performed to ensure that the colonies selected were *Bifidobacteria *[19]. To identify the isolated *Bifidobacterium *spp. at the species level, 16S rRNA sequencing was performed by Bio leaders (Daejeon, Korea). We established an *N*-methyl-*N*'-nitro-*N*-nitrosoguanidine (MNNG)-induced mutant of *B. adolescentis *SPM1005, which we named SPM1005-A. *B. adolescentis *SPM1005-A, was cultured at 37°C for 48 h on general anaerobic medium (GAM, Nissui Pharm, Japan) under anaerobic conditions and then centrifuged at 1,200 rpm for 15 minutes. The supernatant was separated from the bacterial cell pellet and filtered using an 0.2-μm syringe filter (Sartorius Stedim Biotech, Germany). The purified supernatant was used for further experiments. Written informed consents were obtained from all volunteer who provided samples and the protocol was approved by the Institution Review Board of Office of Research Development, Sahmyook University.

### Cell culture and treatment

SiHa cervical cancer cells expressing HPV type 16 were obtained from the American Type Culture Collection http://www.atcc.org. The cell lines were grown in Minimum essential medium alpha (Gibco) containing 10% heat-inactivated fetal bovine serum (Sigma, USA), 10,000 U/ml penicillin and 10,000 μg/ml streptomycin in a humidified incubator at 37°C with 5% CO_2_. SiHa cells (1.0 × 10^5 ^cells per flask) were incubated for 0, 24, or 48 h in the presence or absence of *B. adolescentis *SPM1005-A at a concentration of 5.1 × 10^7 ^cfu/ml. After incubation, the culture medium was removed and the monolayers were washed with phosphate-buffered saline (PBS) without phenol red and supplements; the cells were then immediately used for total protein and RNA extraction.

### Cell viability assay

Cell viability was determined by a Trypan blue dye exclusion assay. Cells (3.6 × 10^5^) in culture plates were incubated overnight, and then the medium was changed to new medium either with or without *B. adolescentis *SPM1005-A at a concentration of 5.1 × 10^7 ^cfu/ml, and incubated for 0, 24, 48, and 72 h. After incubation, each cell suspension was mixed with an equal volume of 0.3% Trypan blue solution (Samchun Chemical, Korea). Finally, cells were observed under a microscope, and living cells were counted on hemocytometer. Morphological changes were observed using an inverted microscope (Olympus, Japan) at × 400 magnification. Each assay was performed three times in triplicate and results presented as % of control.

### Reverse transcription and quantitative real-time PCR (qRT-PCR)

Total RNA was extracted from the cell line using RNeasy mini Kit (Qiagen, USA) according to the manufacturer's recommendation. RNA concentration was quantified by measuring the absorbance at 260 nm. In order to analyze the same amount of cDNA in every sample, we performed reverse transcription as a separate step from PCR. The mRNA was reverse transcribed into cDNA using oligo-dT primer and Omniscript reverse transcription kit (Qiagen). The cDNA was stored at -20°C or directly used in qRT-PCR. As growth of HPV-transformed cervical cancer cells is dependent on sustained viral oncogene E6 and E7 expression, we investigated the inhibitory effects of *B. adolescentis *SPM1005-A on the cervical carcinoma cell line, SiHa, with quantitative real-time PCR (qRT-PCR), which is an excellent method for quantitation of viral DNA [20,21].

qRT-PCR was conducted in a Light Cycler (Roche) system and the data were analyzed with Light Cycler software version 4.5. The quantity of HPV16-E6 and HPV16-E7 transcripts in each sample was standardized to glyceraldehyde 3-phosphate dehydrogenase (GAPDH) transcript levels. For absolute quantification, cDNA constructed with specific binding sites for HPV16 E6 and E7 primers were used to derive the standard curves. Reactions contained 2 μl each of the cDNA solution, MgCl_2_, primers and Fast Start DNA Master SYBR Green I (Qiagen). The primer sequences used for qRT-PCR of HPV16-E6 were 5'-GAC CCA GAA AGT TAC CAC AG-3' (nucleotide 44 to nucleotide 64) and 5'-CAT AAA TCC CGA AAA GCA AAG-3' (nucleotide 153 to nucleotide 173), and of E7 were 5'-GGA GGA GGA TGA AAT AGA TGG-3' (nucleotide 99 to nucleotide 199) and 5'-TGA GAA CAG ATG GGG CAC AC-3' (nucleotide 268 to nucleotide 287). GAPDH was used as internal control with primer sequences of 5'-CTG CAC CAC CAA CTG CTT AG-3' (forward) and 5'-TTC TGG GTG GCA GTG ATG-3' (reverse) [22]. The amplification conditions were initial incubation at 95°C for 10 minutes, followed by 45 cycles of 95°C for 10 s, 60°C for 10 s and 72°C for 10 s. Results were calculated using the ΔΔCT method with cDNA from all samples as a reference as described previously [23]. Positive and negative controls were included in each experiment to ensure reproducible results.

### Protein electrophoresis and immunoblotting

After 24 or 48 h of treatment, each sample was washed with PBS and treated with lysis buffer (Thermo, USA). The samples, containing 30 μg of protein, were suspended in a 2 × sample buffer (Sigma, USA), boiled for 4 minutes and resolved on 15% sodium dodecyl sulfate polyacrylamide gel electrophoresis (SDS-PAGE) gels. The proteins were transferred to nitrocellulose membrane (Whatman, USA), and equal loading was verified with Ponceau S staining (Sigma). Immunodetection was performed with HPV16-E6 antibody and HPV16-E7 antibody (Santa Cruz Biotechnology, USA), followed by incubation with donkey anti-goat horseradish-peroxidase-conjugated antibody and detection using enhanced chemiluminescence western blotting detection reagents (Abfrontier). β-Actin was used as a normalization standard. Each blot is representative of three experiments.

### Statistical analysis

Results were expressed as mean ± SD. One-tailed Student's t tests were employed whenever individual data points were compared. Mean values of *P *< 0.05 were considered statistically significant.

## Results

### Effect of *B. adolescentis *SPM1005-A on morphology

**Figure 1:**
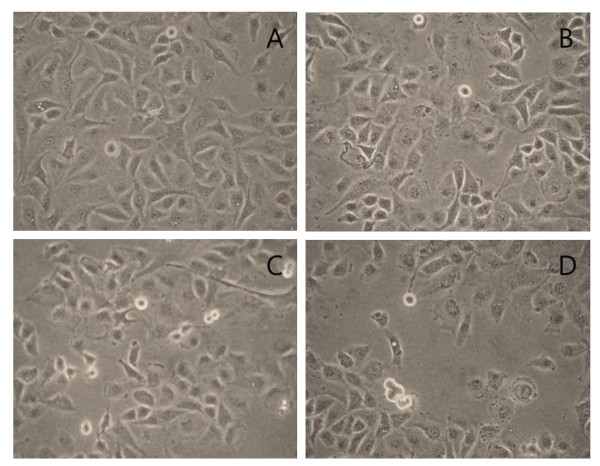
**Morphological changes by *Bifidobacterium adolescentis *SPM1005-A treatment in the cervical cancer cell line SiHa**. **(A) **Phosphate-buffered saline (PBS)-treated SiHa cells were cultured with *B. adolescentis *SPM1005-A at a concentration of 5.1 × 10^7 ^cfu/ml for 24 h **(B)**, 48 h **(C) **and 72 h **(D)**. Cells were viewed under a microscope and photographed at × 400.

Representative images of the morphological changes observed after 24, 48, or 72 h are shown in Figure 1. Compared to control PBS-treated cells, cells exposed to *B. adolescentis *SPM1005-A for 72 h slowly shrank in appearance, but there was no observed difference in 24-h to 72-h treated cells.

### Cytotoxic effects

**Figure 2:**
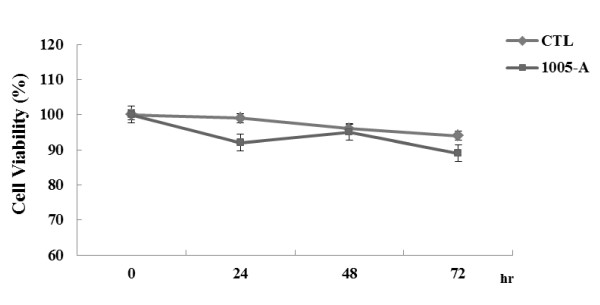
**Effect of *Bifidobacterium adolescentis *SPM1005-A on viability of SiHa cells**. Cells at a density of 1 × 10^7 ^were plated and grown in the presence (squares) of 5.1 × 10^7 ^cfu/ml *B. adolescentis *SPM1005-A or absence (diamonds) for 0, 24, 48, and 72 h. After incubation, cells were split and counted by the Trypan blue dye exclusion assay.

The cytotoxicity of *B. adolescentis *SPM1005-A was evaluated by Trypan blue dye exclusion assay. Based on the results of the assay, the cell survival rate decreased slightly (92, 95, 89%, respectively) with 24-h to 72-h exposure to 5.1 × 10^7 ^cfu/ml *B. adolescentis *SPM1005-A (Figure 2). This indicates that *B. adolescentis *SPM1005-A does not significantly affect the cytotoxicity of SiHa cells. Consequently, experiments to assess the antiviral activity were carried out at this tested concentration in this study.

### Inhibition of *B. adolescentis *SPM1005-A on E6 and E7 expression

**Figure 3:**
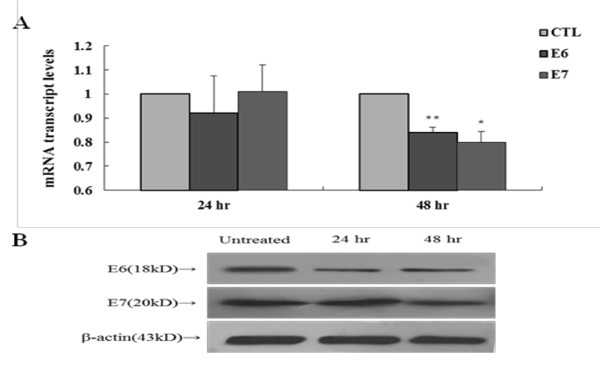
**The expression of E6 and E7 mRNA transcript levels (A) and protein (B) in SiHa cells**. SiHa cells were treated with 5.1 × 10^7 ^cfu/ml *Bifidobacterium adolescentis *SPM1005-A for 24 or 48 h. Transcript levels were standardized against the glyceraldehyde 3-phosphate dehydrogenase (GAPDH) mRNA level as normalization control. Each sample was evaluated by separate experiments, and results represent the means and standard deviation (*P < 0.05, **P < 0.01). The cells were collected and the extracts (30 μg) were resolved on 15% sodium dodecyl sulfate polyacrylamide gel electrophoresis (SDS-PAGE), transferred to nitrocellulose membranes, and immunoblotted with anti-human papillomavirus (HPV)16 E6 and anti-HPV16 E7 polyclonal antibodies. β-Actin was used as a normalization standard. The arrow indicates the location of the protein in the gel.

For this purpose, total RNA was converted to cDNAs for E6, E7 and GAPDH as a control by using oligo-dT primer reverse transcription. The qRT-PCR results showed a reduction of both genes mRNA transcript levels after incubating the cells with *B. adolescentis *SPM1005-A for 24 and 48 h (Figure 3A). PBS-treated cells were used as a control. A reduction in E6 mRNA expression was observed compared with that in untreated cells after 24 h. In particular, E6 and E7 expression levels were significantly inhibited by *B. adolescentis *SPM1005-A treatment for 48 h (*P < 0.05, **P < 0.01).

### Western blotting

Western blot analysis revealed that the expression of both proteins decreased slightly after incubation with *B. adolescentis *SPM1005-A (Figure 3B). Inhibition of E6 protein levels was observed at both 24 and 48 h in comparison to β-actin. The qRT-PCR results revealed that E7 mRNA levels decreased after treatment in the SiHa cell line, whereas the level of E7 protein expression in cells treated with *B. adolescentis *SPM1005-A appeared to be unaffected compared with that in the control. In this respect, the protein change did not consistently occur in a time-dependent manner.

## Discussion

Cervical cancer is the second most common malignant disease of the female and is caused by persistent infection with HPV. The majority of cases of cervical cancer develop as squamous cell carcinomas (SCCs) or adenocarcinoma [24]. The prevalence of HR HPV infection among young women is typically around 20% to 40% depending on geographical region, with the incidence lowering with age as infections are controlled by the host immune system [25,26].

Probiotics, particularly *Bifidobacteria*, are used in fermented food and are widely assumed to be safe. Few attempts have been made recently to study the potential benefits of LAB. *Lactobacillus rhamnosus *GG and *Lactobacillus acidophilus *CRL431 are known to interfere with virus-induced pathology indirectly, by favoring cellular homeostasis, either stimulating innate or adaptive immunity [27]. In addition, several studies have shown that orally delivered probiotics can develop a mutually advantageous symbiosis with the gastrointestinal tract and activate immune systems by the release of proinflammatory cytokines such as tumor necrosis factor (TNFα), interleukin (IL)-12, or IL-6, and by production of anti-inflammatory cytokines such as transforming growth factor β (TGFβ) and IL-10 [28-31]. Some *Bifidobacterium *species have been reported to prevent infections by pathogenic bacteria such as *Escherichia coli, Salmonella *and *Helicobacter pylori *[32-34]. Despite literature indicating the beneficial effects of *Bifidobacteria*, the antiviral effects have not yet been studied in cervical carcinoma cell lines. The antiviral effects of *Bifidobacteria *have rarely been studied in cervical cancer cell lines or *in vivo*, so the molecular mechanisms that underlie these effects need to be investigated in detail.

In the current study, we assessed the inhibitory effects on HPV oncogene mRNA and protein expression. As previously mentioned, the carcinogenesis process of cervical cancer is associated with the overexpression of the viral oncogenic proteins E6 and E7 that inactivate the tumor suppressors, p53 and pRb, block apoptosis, shorten telomeres and reduce immune recognition [35]. There have been several attempts to suppress these two genes typical to HR-HPV16 and HR-HPV18. Li *et al. *demonstrated that oncogenic gene downregulation mechanisms that increase tumor suppressor factors and induce apoptosis may be employed in cervical cancer cells [36].

Our results showed that *B. adolescentis *SPM1005-A can downregulate expression of both genes at both the mRNA and protein levels in SiHa cells. In particular, expression of both genes was decreased significantly with *B. adolescentis *SPM1005-A treatment for 48 h. In addition, decreased HPV16 E6 and E7 gene expressions and protein levels were not associated with cell morphology and significant cytotoxic effects of *B. adolescentis *SPM1005-A in SiHa cells. However, it has been not determined how *B. adolescentis *SPM1005-A regulates the expression of E6 and E7 genes or what is the specific target region.

## Conclusions

In summary, *B. adolescentis *SPM1005-A was found to have antiviral activity through suppression of E6 and E7 oncogene expression. The results suggest that *B. adolescentis *SPM1005-A could be useful for prevention of HPV-associated cervical cancer.

## Competing interests

The authors declare that they have no competing interests.

## Authors' contributions

MKC conceived and designed the experiments. DKL, HMA, and SWL performed the experiments. SHS, JHK and KJK analyzed the data. NJH supervised the study. All authors reviewed and approved the final manuscript.

## Pre-publication history

The pre-publication history for this paper can be accessed here:

http://www.biomedcentral.com/1741-7015/10/72/prepub
